# Long noncoding RNA H19 upregulates vascular endothelial growth factor A to enhance mesenchymal stem cells survival and angiogenic capacity by inhibiting miR-199a-5p

**DOI:** 10.1186/s13287-018-0861-x

**Published:** 2018-04-19

**Authors:** Jingying Hou, Lingyun Wang, Quanhua Wu, Guanghui Zheng, Huibao Long, Hao Wu, Changqing Zhou, Tianzhu Guo, Tingting Zhong, Lei Wang, Xuxiang Chen, Tong Wang

**Affiliations:** 1 Guangdong Provincial Key Laboratory of Malignant Tumor Epigenetics and Gene Regulation, Sun Yat-sen Memorial Hospital of Sun Yat-sen University, 107 Yanjiang Xi Road, Guangzhou, 510120 Guangdong China; 2 Guangdong Province Key Laboratory of Arrhythmia and Electrophysiology, 107 Yanjiang Xi Road, Guangzhou, Guangdong China; 3 Department of Emergency, Sun Yat-sen Memorial Hospital of Sun Yat-sen University, 107 Yanjiang Xi Road, Guangzhou, Guangdong China; 4 Department of Gastroenterology, Sun Yat-sen Memorial Hospital of Sun Yat-sen University, 107 Yanjiang Xi Road, Guangzhou, Guangdong China

**Keywords:** Long noncoding RNA H19, Vascular endothelial growth factor A, Mesenchymal stem cells, MiR-199a-5p, Survival, Angiogenic capacity

## Abstract

**Background:**

Currently, the overall therapeutic efficiency of mesenchymal stem cells (MSCs) transplantation for the treatment of cardiovascular disease is not satisfactory. The low viability and angiogenic capacity of the implanted cells in the local infarct tissues restrict their further application. Evidence shows that long noncoding RNA H19 (lncRNA-H19) mediates cell survival and angiogenesis. Additionally, it is also involved in MSCs biological activities. This study aimed to explore the functional role of lncRNA-H19 in MSCs survival and angiogenic capacity as well as the underlying mechanism.

**Methods:**

MSCs were obtained from C57BL/6 mice and cultured in vitro. Cells at the third passage were divided into the following groups: MSCs+H19, MSCs+H19 NC, MSCs+si-H19, MSCs+si-H19 NC and MSCs. The MSCs+H19 and MSCs+H19 NC groups were transfected with lncRNA-H19 and lncRNA-H19 scramble RNA respectively. The MSCs+si-H19 and MSCs+si-H19 NC groups were transfected with lncRNA-H19 siRNA and lncRNA-H19 siRNA scramble respectively. MSCs were used as the blank control. All groups were exposed to normoxia (20% O_2_) and hypoxia (1% O_2_)/serum deprivation (H/SD) conditions for 24 h. Cell proliferation, apoptosis and vascular densities were assessed. Bioinformatics and dual luciferase reporter assay were performed. Relevant biomarkers were detected in different experimental groups.

**Results:**

Overexpression of lncRNA-H19 improved survival and angiogenic capacity of MSCs under both normoxia and H/SD conditions, whereas its knockdown impaired cell viability and their angiogenic potential. MicroRNA-199a-5p (miR-199a-5p) targeted and downregulated vascular endothelial growth factor A (VEGFA). MiR-199a-5p was a target of lncRNA-H19. LncRNA-H19 transfection led to a decreased level of miR-199a-5p, accompanied with an elevated expression of VEGFA. However, both miR-199a-5p and VEGFA presented inverse alterations in the condition of lncRNA-H19 knockdown.

**Conclusions:**

LncRNA-H19 enhanced MSCs survival and their angiogenic potential in vitro. It could directly upregulate VEGFA expression by inhibiting miR-199a-5p as a competing endogenous RNA. This mechanism contributes to a better understanding of MSCs biological activities and provides new insights for cell therapy based on MSCs transplantation.

## Background

Stem cell transplantation has emerged as a novel therapeutic approach for the treatment of cardiovascular disease [1]. Numerous studies reveal that stem cell transplantation results in cardiomyocyte differentiation and neovascularization [2]. Mesenchymal stem cells (MSCs) have been considered as an optimal source in cell therapy [3, 4]. MSCs can differentiate into angioblasts, including endothelial cells (ECs) and vascular smooth muscle cells [4]. However, inferior survival ability and low transdifferentiation efficiency of these cells abrogate their therapeutic efficacy [5, 6]. The newly formed vascular densities are sparse in the local infarct region after MSCs transplantation [5, 7]. In view of this, it is imperative to excavate the relevant molecular mechanisms that dominate their survival and angiogenic capacity.

The rapid development of genome sequencing technologies has brought various types of noncoding RNAs (ncRNAs) that serve as pivotal components of gene regulatory networks to the forefront [8]. Long ncRNAs (lncRNAs) are more than 200 nucleotides in length and represent the most prevalent and novel class of ncRNA molecules. Recent data show that lncRNAs can act as regulators in multiple biological processes, including stem cell lineage specification and differentiation [9]. In addition, they are also implicated in the modulation of vascular function and angiogenesis [10–12].

The long noncoding RNA H19 (lncRNA-H19), which is 2.3 kb in length and located in chromosome 11, is a nonprotein-coding imprinted and maternally expressed lncRNA [13]. Evidence indicates that lncRNA-H19 facilitates MSCs proliferation [14]. In addition, it also executes regulatory roles in their lineage differentiation [15, 16]. Moreover, lncRNA-H19 has been demonstrated to enhance angiogenesis in hypoxic conditions [17]. However, it remains unclear whether it is involved in the regulation of MSCs angiogenic capacity and what might be the underlying mechanism. In this study, we tried to investigate the role of lncRNA-H19 in MSCs survival and angiogenic capacity in vitro and explored the relevant mechanism.

## Methods

### Ethics statement

Three-week-old C57BL/6 mice were obtained from the Animal Experimental Center of Sun Yat-sen University. All animal handling and procedures were performed in accordance with protocols approved by the Animal Ethics Committee of Sun Yat-sen University (201702001).

### Isolation and culture of MSCs

Bone marrow cells were collected from C57BL/6 mice. Isolation and culture of MSCs were performed as reported previously [18, 19]. Characteristics of MSCs were identified by fluorescence-activated cell sorting (FACS) as reported previously [5, 18, 19]. MSCs were positive for CD44 and CD29, but negative for CD34. Third-passage MSCs were used for all of the experiments.

### Plasmid construction

For overexpression of H19, the following primers were used for amplification: H19 forward, 5′-CCGGAATTCACCGGGTGTGGGAGGGGGGTGGGGGGT-3′; and H19 reverse, 5′-CCGCTCGAGATGACTGTAACTGTATTTATTGATGG-3′. H19 cDNA products were cloned in the mammalian expression pcDNA3.1 vector (Invitrogen) at sites *Eco*RI and *Xho*I (TaKaRa, Dalian, China) and plasmid carrying a nontargeting sequence was used as a negative control. To knock down H19 expression, three complementary oligonucleotides of siRNA (H19-siRNA1#, 5′-CCGUAAUUCACUUAGAAGAdTdT-3′; H19-siRNA2#, 5′-CACAUAGAAAGGCAGGAUAdTdT-3′; and H19-siRNA3#, 5′-CCUUCUAAACGAAGGUUUAdTdT-3′) and a scramble negative siRNA (5′-UUCUCCGAACGUGUCACGUTT-3′) were constructed. The interfering sequence with the highest inhibition efficiency was screened and used in the subsequent experiments.

### Transient transfection experiments

MSCs were incubated at 1 × 10^6^ cells per well in six-well plates. pc3.1-H19, pc3.1-H19 scramble negative control (H19 NC), H19 siRNA(si-H19), scramble negative control of H19 siRNA (si-H19 NC), miR-199a-5p mimics, miR-199a-5p mimics negative control (miR-199a-5p NC), miR-199a-5p inhibitor and miR-199a-5p inhibitor negative control (miR-199a-5p inhibitor NC) were transiently transfected into MSCs. Transfection of MSCs was performed by using Lipofectamine2000 (Invitrogen) according to the manufacturer’s instructions. LncRNA-H19 overexpression or knockdown was determined by quantitative real-time polymerase chain reaction (qRT-PCR).

### Hypoxia/serum deprivation treatment of MSCs

The hypoxia/serum deprivation (H/SD) model was used to mimic the ischemic environment. MSCs in different experimental groups were incubated in normoxia (20%O_2_) and H/SD (serum-free media with 1% O_2_) conditions in a Galaxy® 48 R incubator (Eppendorf/Galaxy Corporation, USA) at 37 °C for 24 h as described previously [5, 18]. Three cultures were used in each group.

### Proliferation and apoptosis evaluation of MSCs

After the aforementioned treatments, the MSCs of different groups were collected and suspended in complete culture medium. The MTS assay (cellTiter96AQ, one solution cell proliferation assay, catalog number G3582; Promega, Madison, WI, USA) was applied to evaluate cell survival and proliferation as reported previously [5, 18]. The terminal deoxynucleotidyl transferase biotin-dUPT nick end-labeling (TUNEL) assay was performed to detect MSCs apoptosis after normoxia and H/SD exposure for 24 h in vitro as reported previously [5, 18]. All sections were examined and the apoptosis rate was calculated by randomly selecting five different areas under a florescent microscope (DMI6000B; Leica, Brunswick, Germany) [5, 18].

### Tube formation assay

Aliquots of human umbilical vein endothelial cells (HUVECs) (Yiyuan Biotechnology Corporation, GuangZhou, China) at a concentration of 2 × 10^4^ cells per well were seeded onto Matrigel-coated wells (catalog number 356234; BD Corporation) of a 24-well plate. Tube formation assay was performed as reported previously [18]. The numbers of the vascular branches (formation of closed structures of HUVECs) were examined and calculated by randomly selecting five different fields per well in triplicate experiments by using a phase-contrast microscopy (CKX41, U-CTR30-2; OLYMPUS Japan) as described previously [5, 18].

### Vector construction and luciferase reporter assay

Two luciferase reporters containing wild-type H19 (psiCHECK2-H19-WT, which encompassed the binding sites for miR-199a-5p) or mutant H19 (psiCHECK2-H19-MU, which encompassed the mutant sequence of the binding sites for miR-199a-5p) were constructed to validate the interaction between H19 and miR-199a-5p. H19 (containing the binding sites for miR-199a-5p) was amplified with the following primer sequences: forward, 5′-CCGCTCGAGACCGGGTGTGGGAGGGGGGTGGGGGGT-3′; and reverse, 5′-ATAAGAATGCGGCCGCATGACTGTAACTGTATTTATTGATG-3′. Mutant H19 contained a mutation site eliminating targeting by miR-199a-5p, and its primer sequences were as follows: forward, 5′-GCGGAAAGGGCCCACAGTGGACTTGAGCTCTGATATGCCCTAACCGCTCAGTCCCTGG-3′; and reverse, 5′-CCAGGGACTGAGCGGTTAGGGCATATCAGAGCTCAAGTCCACTGTGGGCCCTTTCCGC-3′.

Cells were seeded in 24-well plates and cotransfected with wild-type or mutated luciferase construct along with miR-199a-5p mimics or miR-199a-5p mimics negative control. After 48 h of transfection, measurement was performed with the dual-luciferase reporter assay system (Promega). The relative luciferase activity was calculated by the ratio of firefly luciferase activity to renilla luciferase activity.

### Western blot analysis

Protein levels were measured by western blot as reported previously [5, 18, 19]. After washing several times with PBS, cells were collected and lysed with modified RIPA buffer. Cells were completely lysed after repeated vortexing. Supernatants were obtained though centrifugation at 14,000×*g* for 20 min. Proteins were resolved by sodium dodecyl sulfate polyacrylamide gel (SDS-PAGE) and transferred to a polyvinylidenedifluoride (PVDF) membrane (IPVH00010; Millipore, Boston, MA, USA) before incubating with the primary anti-VEGFA rabbit polyclone antibody (Abcam, UK) overnight at 4 °C. The membranes were incubated with anti-IgG horseradish peroxidase-conjugated secondary antibody (Southern Biotech, Birmingham, AL, USA) for 60 min at room temperature following three 5-min washes with TBST. The bands were detected by enhanced chemiluminescence after extensive washing, and band intensities were quantified using image software (image J 2×, version 2.1.4.7) [5, 18, 19].

### qRT-PCR analysis

qRT-PCR was performed as reported previously [5, 18, 19]. Total RNA was isolated from cells using a Trizol reagent (Invitrogen) and digested with RNase-free DNase (Promega) afterward. Concentration and integrity of total RNA were detected and the RT-PCR was conducted on a Sequence Detection System (ABI PRISM®7500) using SYBR Green qPCR SuperMix (Invitrogen). The primers are presented in Table 1. Specific products were amplified and examined (Applied Biosystems) at 95 °C for 10 min, followed by 40 cycles at 95 °C for 15 s and at 60 °C for 30 s, at which point data were acquired. The 2^−ΔΔCt^ method was used for the calculation of the relative level of mRNA. The values for the MSCs group were taken as a base unit under both normoxia and H/SD conditions. The results were quantified as the threshold cycle of each target gene and normalized into the ΔCt value. Quantifications of fold-change in gene expressions were also performed by using the 2^−ΔΔCt^ method [5, 18, 19].

**Table 1:** Primers for quantitative real-time polymerase chain reaction

Gene name	Forward primer (5′–3′)	Reverse primer (5′–3′)
*H19*	GCTCCACTGACCTTCTAAAC	ACGATGTCTCCTTTGCTAAC
*MiR-199a-5p*	ACACTCCAGCTGGGCCCAGTGTTCAGACTACCT	CTCAACTGGTGTCGTGGA
*VEGFA*	AGATTCTGCAAGAGCACC	AAGGTCCTCCTGAGCTAT
*U6*	CTCGCTTCGGCAGCACA	AACGCTTCACGAATTTGCGT
*β-actin*	GCTTCTAGGCGGACTGTTAC	CCATGCCAATGTTGTCTCTT

### Statistical analysis

All quantitative data were described as mean ± SD. The significance of differences among groups was determined by analysis of variance and Scheffe’s multiple-comparison techniques. The methods and assays were repeated three times to ensure the accuracy of the results. Comparisons between time-based measurements within each group were performed with analysis of variance for repeated measurements. *P* < 0.05 was considered statistically significant.

## Results

### Interference efficiency of H19 siRNA and the expression of lncRNA-H19 in different conditions

**Fig. 1:**
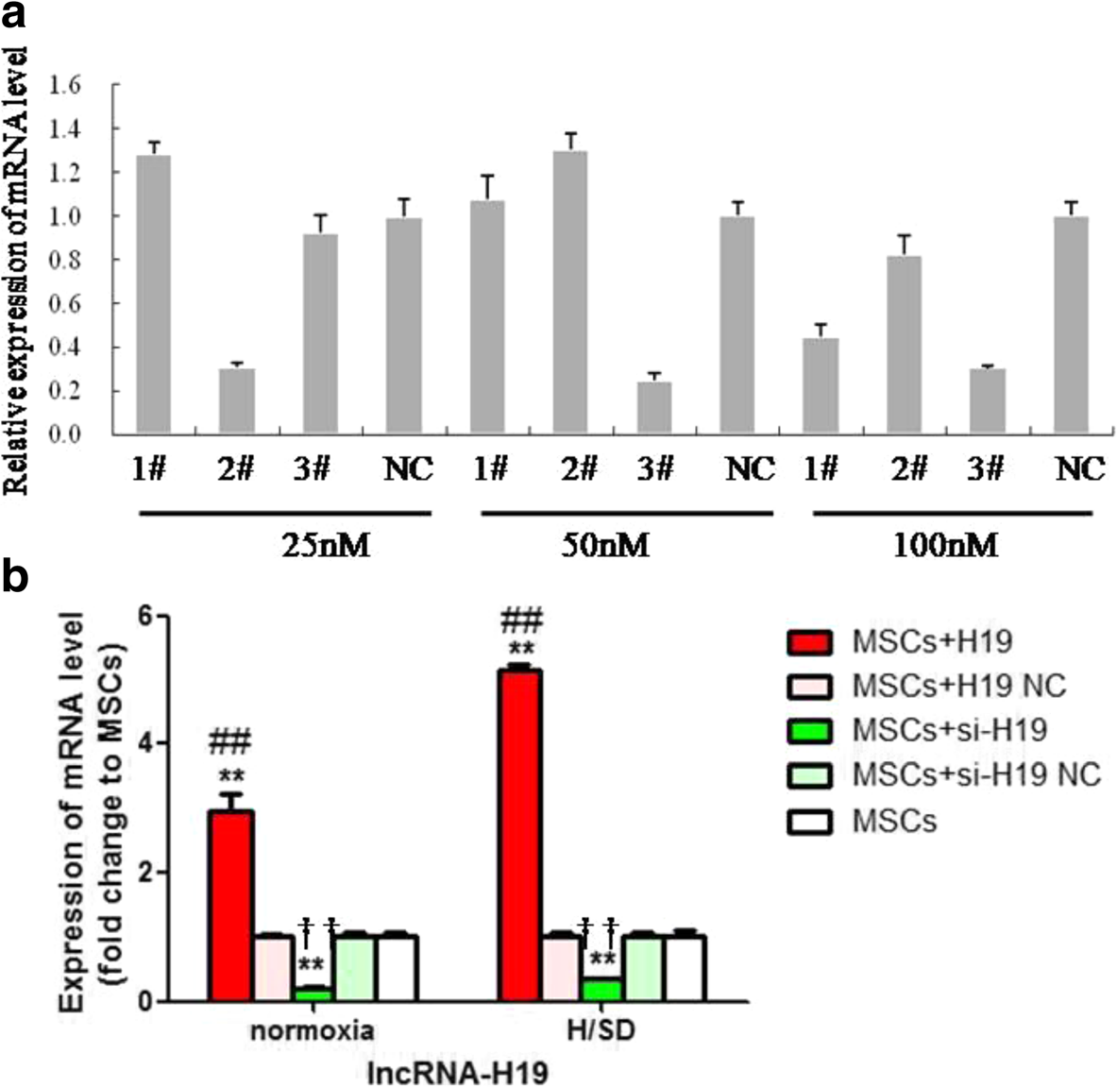
Interference efficiency of H19 siRNA and expression of lncRNA-H19 in different conditions. **a** To evaluate interference efficiency of H19 siRNA, mRNA expression of lncRNA-H19 was detected using qRT-PCR. 1#, 2# and 3# represented H19-siRNA1#, H19-siRNA2# and H19-siRNA3# respectively. Transfection concentration of siRNA interference sequences were designed as 25 nM, 50 nM and 100 nM respectively. **b** qRT-PCR analysis of lncRNA-H19 expression in normoxia and H/SD conditions. MSCs+H19, MSCs transfected with lncRNA-H19; MSCs+H19 NC, MSCs transfected with lncRNA-H19 scramble; MSCs+si-H19, MSCs transfected with lncRNA-H19 siRNA; MSCs+si-H19 NC, MSCs transfected with lncRNA-H19 siRNA scramble. **P < 0.01 vs MSCs, ##*P* < 0.01vs MSCs+H19 NC, ††*P* < 0.01 vs MSCs+si-H19 NC. H/SD: hypoxia/serum deprivation, lncRNA-H19: long noncoding RNA H19, MSCs: mesenchymal stem cells, NC: scramble negative control

Interference efficiency of H19 siRNA was evaluated by expression of the mRNA level of lncRNA-H19. It was revealed that H19-siRNA3# with a concentration of 50 nM exhibited the highest inhibition efficiency and this was used in subsequent experiments (Fig. 1a). The crucial role of lncRNA-H19 in the regulation of cell survival and angiogenic potential was verified by targeted gene overexpression or silencing studies. LncRNA-H19 expression status was confirmed by qRT-PCR analysis. It was shown that the mRNA level of lncRNA-H19 was distinctly increased in the MSCs+H19 group compared with the MSCs+H19 NC and MSCs groups under both normoxia and H/SD conditions, while its expression level was significantly decreased in the MSCs+si-H19 group in contrast with the MSCs+si-H19 NC and MSCs groups (*P* < 0.01, Fig. 1b), indicating that lncRNA-H19 and its siRNA were successfully transfected into MSCs.

### LncRNA-H19 promoted MSCs proliferation

**Fig. 2:**
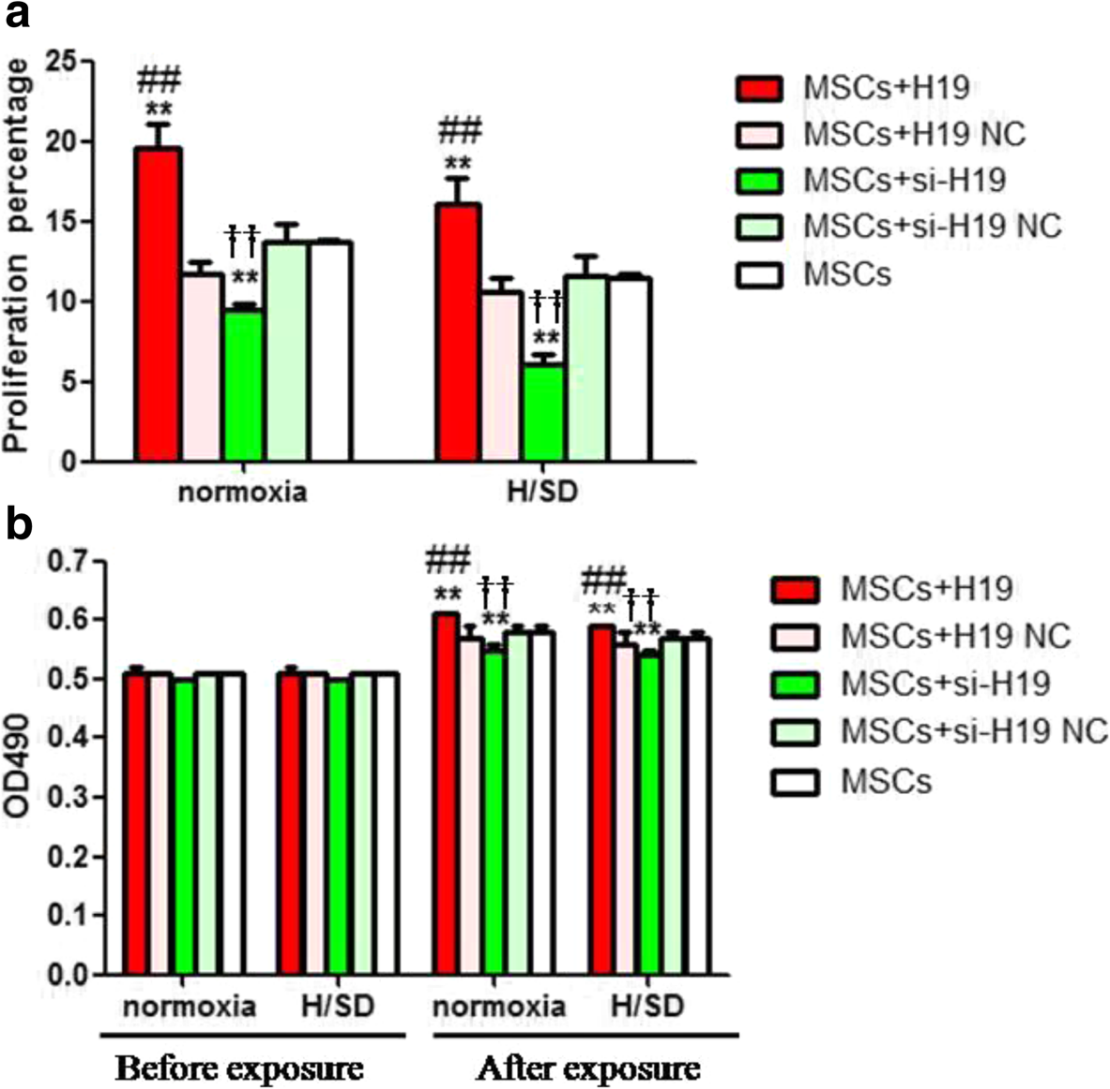
LncRNA-H19 promoted MSCs proliferation. **a** Proliferation rates in different experimental groups under normoxia and H/SD conditions. **b** Optical density (OD) values in different experimental groups under normoxia and H/SD conditions. Proliferation rate = (OD value ​​after exposure / OD value before exposure – 1) × 100% (same sample). MSCs+H19, MSCs transfected with lncRNA-H19; MSCs+H19 NC, MSCs transfected with lncRNA-H19 scramble; MSCs+si-H19, MSCs transfected with lncRNA-H19 siRNA; MSCs+si-H19 NC, MSCs transfected with lncRNA-H19 siRNA scramble. **P < 0.01 vs MSCs, ##*P* < 0.01vs MSCs+H19 NC, ††*P* < 0.01 vs MSCs+si-H19 NC. H/SD: hypoxia/serum deprivation, MSCs: mesenchymal stem cells, NC: scramble negative control

It was revealed that lncRNA-H19 overexpression increased cell proliferation, whereas its knockdown significantly reduced cell proliferation. The MSCs+H19 group exhibited a more rapid growth compared with the MSCs+H19 NC and MSCs groups under both normoxia and H/SD conditions (*P* < 0.01, Fig. 2a, b). However, the MSCs+si-H19 group showed a much lower growth rate in contrast with the MSCs+si-H19 NC and MSCs groups (*P* < 0.01, Fig. 2a, b).

### LncRNA-H19 reduced MSCs apoptosis

**Fig. 3:**
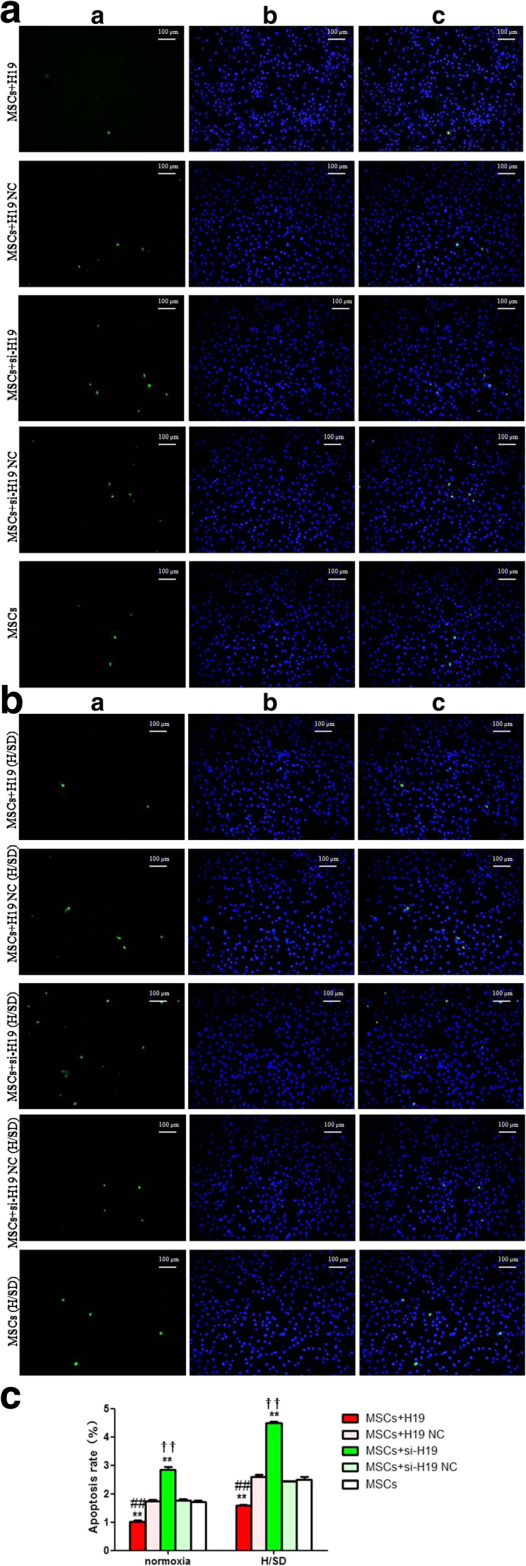
LncRNA-H19 reduced apoptosis of MSCs. **a**, **b** Apoptosis of MSCs by TUNEL staining under normoxia and H/SD conditions respectively. (*a*) TUNEL staining. (*b*) DAPI staining localization. (*c*) Overlap figure of (*a*) and (*b*). **c** Comparison of apoptosic rates among different groups under normoxia and H/SD conditions. MSCs+H19, MSCs transfected with lncRNA-H19; MSCs+H19 NC, MSCs transfected with lncRNA-H19 scramble; MSCs+si-H19, MSCs transfected with lncRNA-H19 siRNA; MSCs+si-H19 NC, MSCs transfected with lncRNA-H19 siRNA scramble. **P < 0.01 vs MSCs, ##*P* < 0.01vs MSCs+H19 NC, ††*P* < 0.01 vs MSCs+si-H19 NC. H/SD: hypoxia/serum deprivation, MSCs: mesenchymal stem cells, NC: scramble negative control

Cell apoptosis was significantly reduced after lncRNA-H19 transfection. The apoptosis rate of the MSCs+H19 group was much lower compared with the MSCs+H19 NC and MSCs groups under both normoxia and H/SD conditions (*P* < 0.01, Fig. 3A, B). However, cell apoptosis was aggravated in the condition of lncRNA-H19 knockdown. There was a distinct increase of the apoptosis rate in the MSCs+si-H19 group in contrast with the MSCs+si-H19 NC and MSCs groups under both normoxia and H/SD conditions (*P* < 0.01, Fig. 3A, B).

### LncRNA-H19 enhanced angiogenic potential of MSCs

**Fig. 4:**
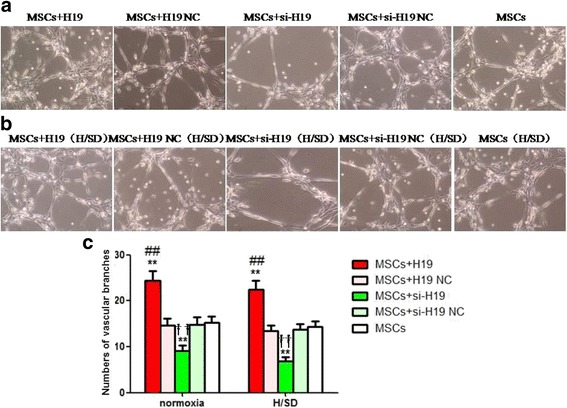
LncRNA-H19 enhanced angiogenic capacity of MSCs. **a**, **b** Results of tube formation assay in different groups under normoxia and H/SD conditions in vitro (magnification ×100). **c** Comparison of numbers of vascular branches among different groups under normoxia and H/SD conditions respectively. MSCs+H19, MSCs transfected with lncRNA-H19; MSCs+H19 NC, MSCs transfected with lncRNA-H19 scramble; MSCs+si-H19, MSCs transfected with lncRNA-H19 siRNA; MSCs+si-H19 NC, MSCs transfected with lncRNA-H19 siRNA scramble. **P < 0.01 vs MSCs, ##*P* < 0.01 vs MSCs+H19 NC, ††*P* < 0.01 vs MSCs+si-H19 NC. H/SD: hypoxia/serum deprivation, MSCs: mesenchymal stem cells, NC: scramble negative control

The number of vascular branches in the MSCs+H19 group was obviously higher than that in the MSCs+H19 NC and MSCs groups under both normoxia and H/SD conditions (*P* < 0.01, Fig. 4a, b). It was shown that there was a much lower number of vascular branches in the MSCs+si-H19 group compared with the MSCs+si-H19 NC and MSCs groups (*P* < 0.01, Fig. 4a, b). These indicated that lncRNA-H19 enhanced angiogenic potential of MSCs.

### LncRNA-H19 upregulated the expression of VEGFA

**Fig. 5:**
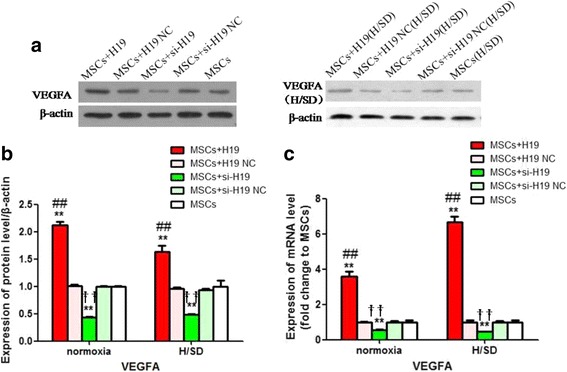
LncRNA-H19 upregulated the expression of VEGFA. Alterations of VEGFA in different experimental groups were detected after lncRAN-H19 transfection or knockdown under normoxia and H/SD conditions. **a, b** Western blot analysis of the protein expression of VEGFA. **c** qRT-PCR analysis of the mRNA expression of VEGFA. MSCs+H19, MSCs transfected with lncRNA-H19; MSCs+H19 NC, MSCs transfected with lncRNA-H19 scramble; MSCs+si-H19, MSCs transfected with lncRNA-H19 siRNA; MSCs+si-H19 NC, MSCs transfected with lncRNA-H19 siRNA scramble. **P < 0.01 vs MSCs,## *P* < 0.01vs MSCs+H19 NC, †† *P* < 0.01 vs MSCs+si-H19 NC. H/SD: hypoxia/serum deprivation, MSCs: mesenchymal stem cells, NC: scramble negative control

As VEGFA is a positive mediator of MSCs survival and angiogenic potential, its protein and mRNA levels were detected in the circumstance of lncRNA-H19 overexpression or knockdown. Western blot and qRT-PCR analyses showed that VEGFA expression was dramatically strengthened in the MSCs+H19 group compared with the MSCs+H19 NC and MSCs groups under both normoxia and H/SD conditions (*P* < 0.01, Fig. 5a, b; *P* < 0.01, Fig. 5c), whereas its expression was prominently attenuated in the MSCs+si-H19 group in contrast with the MSCs+si-H19 NC and MSCs groups (*P* < 0.01, Fig. 5a, b; *P* < 0.01, Fig. 5c). These results revealed that lncRNA-H19 could upregulate the expression of VEGFA.

### MiR-199a-5p negatively regulated VEGFA

**Fig. 6:**
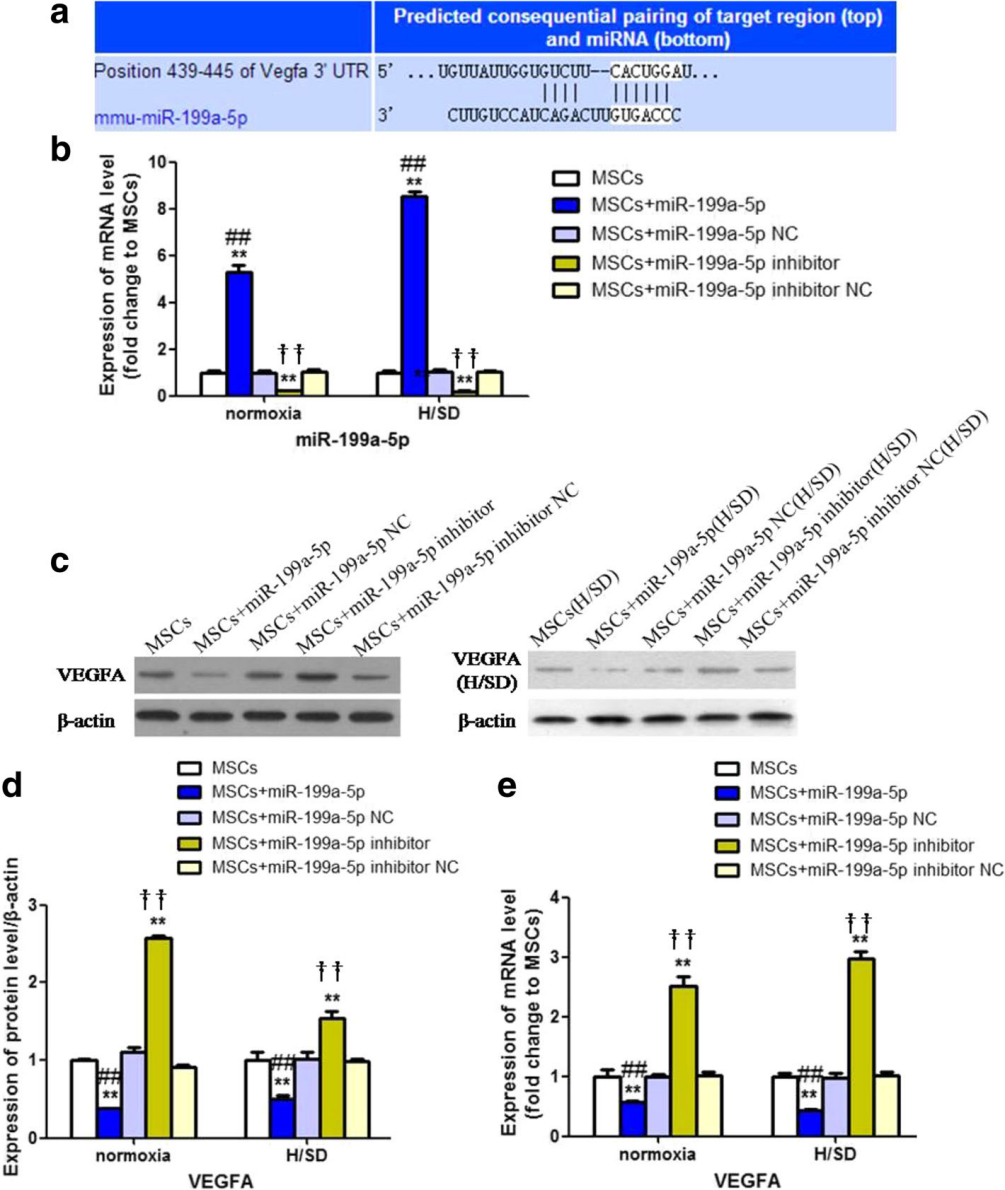
MiR-199a 5p downregulated the expression of VEGFA. Alterations of VEGFA in different experimental groups were detected after miR-199a-5p transfection or knockdown under normoxia and H/SD condtions. **a** Potential consequential paring of VEGFA-targeted region and miR-199a-5p. **b** Expression of miR-199a-5p detected by qRT-PCR. **c, d** Western blot analysis of the protein expression of VEGFA. **e** qRT-PCR analysis of the mRNA expression of VEGFA. MSCs+miR-199a-5p, MSCs transfected with miR-199a-5p mimics; MSCs+miR-199a-5p NC, MSCs transfected with miR-199a-5p mimics negative control; MSCs+miR-199a-5p inhibitor, MSCs transfected with miR-199a-5p inhibitor; MSCs+miR-199a-5p inhibitor NC, MSCs transfected with miR-199a-5p inhibitor negative control. **P < 0.01 vs MSCs, ##*P* < 0.01vs miR-199a-5p NC, ††*P* < 0.01 vs miR-199a-5p inhibitor NC. H/SD: hypoxia/serum deprivation, MSCs: mesenchymal stem cells, NC: scramble negative control, UTR: untranslated region, VEGFA: vascular endothelial growth factor A

VEGFA has been validated as a target of miR-199a-5p and their interaction has been predicted by bioinformatics and confirmed by functional experiments in MSCs in previous studies (Fig. 6a) [20]. To determine whether VEGFA was negatively regulated by miR-199a-5p, its expression was examined in MSCs treated with miR-199a-5p mimics or miR-199a-5p inhibitor. The miR-199a-5p expression status was detected by the qRT-PCR analysis (*P* < 0.01; Fig. 6b). It was shown that the protein and mRNA levels of VEGFA were obviously decreased in the MSCs+miR-199a-5p group under both normoxia and H/SD conditions (*P* < 0.01, Fig. 6c, d; *P* < 0.01, Fig. 6e), while the MSCs+miR-199a-5p inhibitor group presented a distinct augmentation of VEGFA (*P* < 0.01, Fig. 6c, d; *P* < 0.01, Fig. 6e). All these results demonstrated that miR-199a-5p was a negative regulator of VEGFA.

### LncRNA-H19 targeted and negatively regulated miR-199a-5p

**Fig. 7:**
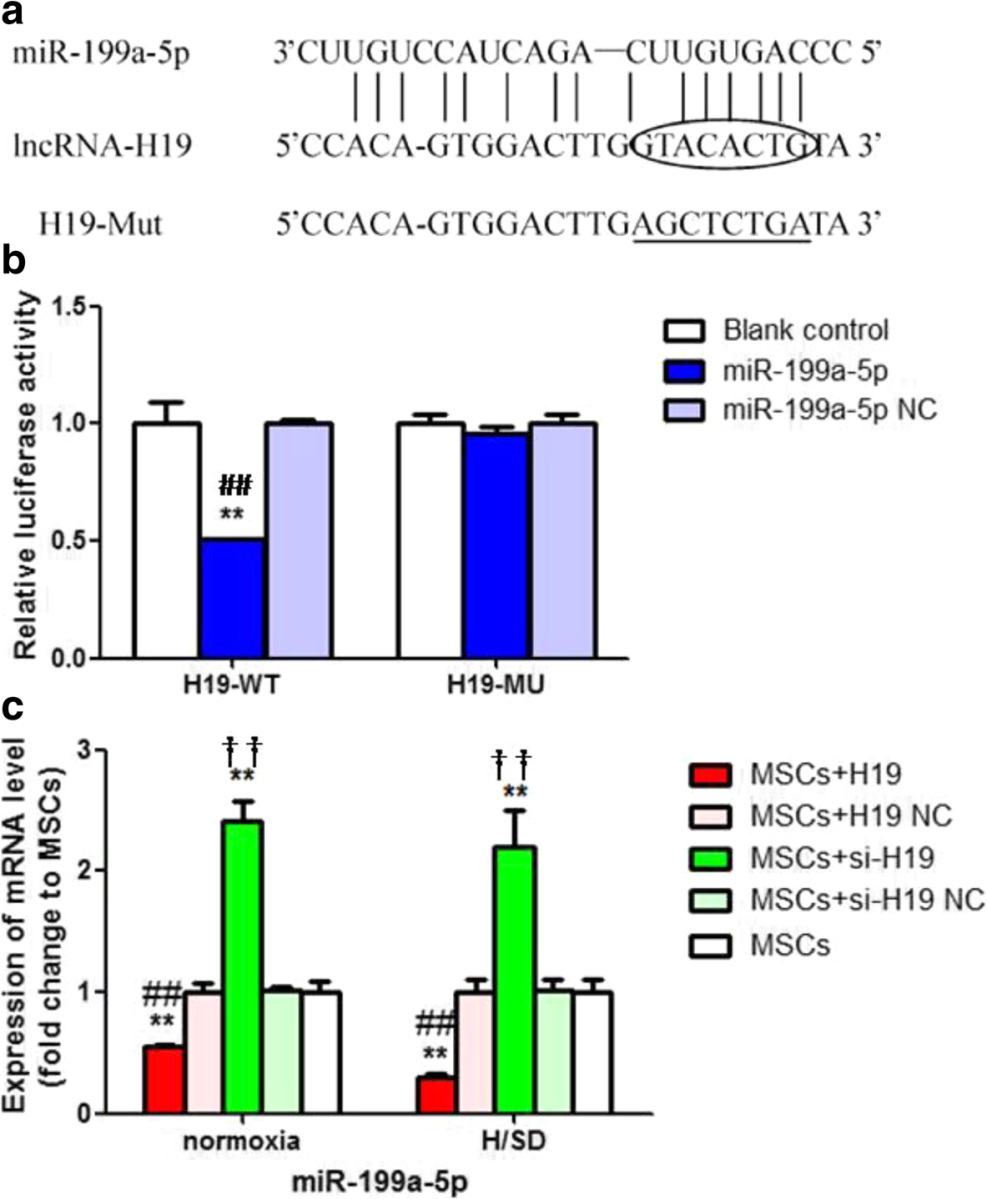
LncRNA-H19 targeted and negatively regulated miR-199a 5p. **a** Potential binding sites of lncRNA-H19 and miR-199a-5p. **b** Results of promega dual-luciferase reporter gene assay. Cells were transiently transfected with luciferase reporter contained wild-type (H19-WT) or mutant (H19-MU) H19. Measurement was performed with dual-luciferase reporter assay system, and luciferase activity was analyzed. Blank control, MSCs transfected with luciferase reporter containing H19-WT or H19-MU; miR-199a-5p, MSCs cotransfected with miR-199a-5p mimics and luciferase reporter containing H19-WT or H19-MU; miR-199a-5p NC, MSCs cotransfected with miR-199a-5p mimics negative control and luciferase reporter containing H19-WT or H19-MU. **c** Alterations of miR-199a-5p after lncRNA-H19 transfection or knockdown under normoxia and H/SD conditions. qRT-PCR were performed to explore expression of miR-199a-5p in different experimental groups under normoxia and H/SD conditions. MSCs+H19, MSCs transfected with lncRNA-H19; MSCs+H19 NC, MSCs transfected with lncRNA-H19 scramble; MSCs+si-H19, MSCs transfected with lncRNA-H19 siRNA; MSCs+si-H19 NC, MSCs transfected with lncRNA-H19 siRNA scramble. **P < 0.01 vs MSCs, ##*P* < 0.01vs MSCs+H19 NC, ††*P* < 0.01 vs MSCs+si-H19 NC. H/SD: hypoxia/serum deprivation, MSCs: mesenchymal stem cells, NC: scramble negative control

LncRNAs can act as a molecular sponge to impede microRNAs (miRNAs) from binding to the mRNA targets and mediate their functions. The potential lncRNA–miRNA mutual binding sites were predicted by the bioinformatics tool (RegRNA2.0, Fig. 7a). We focused on the interaction between lncRNA-H19 and miR-199a-5p, and a dual luciferase reporter assay was performed to further corroborate the specific interplay between lncRNA-H19 and miR-199a-5p. It was indicated that cotransfection of psiCHECK2-H19-WT and miR-199a-5p mimics distinctly diminished the relative luciferase activity in contrast with that in the blank control and miR-199a-5p NC groups (*P* < 0.01, Fig. 7b), while no difference was revealed in the relative luciferase activity between the psiCHECK2-H19-MU+miR-199a-5p and psiCHECK2-H19-MU+miR-199a-5p NC groups (*P* < 0.01, Fig. 7b).

We further examined the expression of lncRNA-H19 and miR-199a-5p in the condition of lncRNA-H19 overexpression or knockdown under both normoxia and H/SD conditions in order to identify whether miR-199a-5p could be regulated by lncRNA-H19. The MSCs+H19 group presented an obvious downregulation of miR-199a-5p compared with the MSCs+H19 NC and MSCs groups (*P* < 0.01, Fig. 7c). Nevertheless, the MSCs+si-H19 group showed a significantly elevated level of miR-199-5p in contrast to the MSCs+si-H19 NC and MSCs groups (*P* < 0.01, Fig. 7c). The aforementioned luciferase reporter assay and the subsequent functional detection confirmed that lncRNA-H19 could competitively inhibit miR-199a-5p to further upregulate the expression of VEGFA.

## Discussion

In the present work, we demonstrated that lncRNA-H19 enhanced MSCs survival and angiogenic capacity by acting as a molecular sponge to competitively inhibit miR-199a-5p and further modulated its target VEGFA.

The H19 gene has been pervasively studied as a model of genomic imprinting although some of its functions remain enigmatic. LncRNA-H19 is involved in the modulation of cell survival. The pro-proliferative capability of lncRNA-H19 has been verified by evidential studies [21, 22]. LncRNA-H19 endows cells with the ability to resist stress and affords a growth advantage. Cytoprotective properties of lncRNA-H19 on progenitor cells have been reported by several previous studies. Upregulation of lncRNA-H19 protects myogenic progenitor cells from death and improves their survival under hypoxia [21]. LncRNA-H19 knockdown cripples the expression of the pluripotency markers in embryonic stem cells and suppresses their proliferation [22] Recent studies manifest that lncRNA-H19 participated in MSCs proliferation and lineage differentiation. LncRNA-H19 maintains a polyploid state of MSCs and facilitates their proliferation [14]. It regulates MSCs transdifferentiation into osteoblast and neural cells [15, 16]. In this study, it was discovered that lncRNA-H19 overexpression could promote MSCs proliferation and diminish cell apoptosis under both normoxia and H/SD conditions in vitro. LncRNA-H19 blockage resulted in cell growth arrest and induced apoptosis. These findings suggested that lncRNA-H19 performed vital roles in enhancing MSCs survival.

LncRNA-H19 participates in vascular physiopathology and angiogenesis.LncRNA-H19 knockdown causes cell cycle arrest of HUVECs and weakens their potential to form capillary-like structures [17]. LncRNA-H19 can mediate phenotype of ECs and triggers angiogenesis [23]. Increased expression of lncRNA-H19 contributes to stemness and angiogenesis of glioblastoma cells [24]. In this study, lncRNA-H19 transfection into MSCs remarkably impelled tube formation under both normoxia and H/SD conditions when media from these cells were cocultured with HUVECs, whereas vascular like structures were damaged once the expression of lncRNA-H19 in MSCs was depressed. These results implied the crucial role of lncRNA-H19 in the regulation of angiogenic potential of MSCs.

LncRNA-H19 could promote MSCs survival and angiogenic capacity, so the specific mechanism was further investigated. VEGF is as a crucial mediator of MSCs survival and angiogenesis. Abundant evidence exhibits that upregulation of VEGF promotes MSCs survival and intensifies their angiogenic potential [5, 18, 25, 26]. LncRNA-H19 may participate in the regulation of VEGF. Expansion of MSCs in an early stage under hypoxic conditions accompanies a synchronized upregulated expression of lncRNA-H19 and VEGF [14]. Some other data indicate that lncRNA-H19 drives cell proliferation and survival, and abates apoptosis by propelling the expression of VEGF [27, 28]. In this study, it was discovered that lncRNA-H19 transfection led to an increased expression of VEGFA, whereas its knockout caused an obvious reduction of VEGFA, supporting that lncRNA-H19 enhanced MSCs survival and angiogenic capacity by upregulating VEGFA.

MiRNAs are highly conserved ncRNAs that exert versatile biological functions [29, 30]. They regulate gene expression at the transcriptional or post-transcriptional level by targeting the 3′-untranslated region (3′-UTR) of genes. Various miRNAs have been certified to be closely linked with MSCs survival and angiogenesis [31, 32]. MiR-199a-5p plays a negative regulatory role in cell survival and angiogenesis. Downregulation of miR-199a-5p in pulmonary microvascular ECs accelerates proliferation and angiogenesis [33]. Enforced expression of miR-199a-5p provokes downregulated expression of proangiogenic factors in hypoxic multiple myeloma cells and impairs the migration of ECs and angiogenesis [34]. VEGFA has been certified as a target of miR-199a-5p [20]. MiR-199a-5p prohibits VEGFA-induced cell proliferation, migration and angiogenesis. It suppresses cell proliferation, motility and angiogenesis of endometrial MSCs by directly targeting the 3′-UTR of VEGFA and inhibiting its expression [20]. Here, we also found that the expression level of VEGFA was significantly receded after the transfection of miR-199a-5p mimics, whereas miR-199a-5p inhibition remarkably raised its level, suggesting that VEGFA was negatively regulated by miR-199a-5p.

The interaction between lncRNA–miRNA functional networks has drawn much attention in recent years [35]. LncRNA-H19 can function as a primary miRNA transcript or as a miRNA sponge [36, 37]. There are already several records of lncRNA-H19 functioning as a “competing endogenous RNA” (ceRNA) to participate in cell proliferation, differentiation and angiogenesis. It has been reported that lncRNA-H19 functions as a ceRNA by acting as a sink for miR-17-5p in the modulation of YES1 expression to prompt thyroid cancer cell proliferation and migration [38]. There is another study showing that lncRNA-H19 sponges and antagonizes let-7 miRNA to control muscle differentiation [39]. One latest study exhibits that lncRNA-H19 inhibits miR-29a to upregulate the angiogenic factor VASH2 and modulate proliferation and tube formation of glioma vascular ECs in vitro [40]. LncRNA-H19 and miR-199a-5p exhibit opposite regulatory effects in a plethora of biological activities of organisms [41–44]. In the present work, to fully understand the functionary mechanism of lncRNA-H19, we speculated that it acted as a sponge for miR-199a-5p to mediate the target gene VEGFA. We explored the potential role of lncRNA-H19 as a ceRNA to inhibit miR-199a-5p. Bioinformatic prediction combined with functional experiments proffered a further sustained interaction between lncRNA-H19 and miR-199a-5p. Dual luciferase report and qRT-PCR assays confirmed that lncRNA-H19 could target miR-199a-5p. Dual luciferase reporter assay indicated that the H19 reporter gene luciferase activity was significantly decreased in miR-199a-5p transfection group. Further qRT-PCR analysis displayed that miR-199a-5p was inversely correlated with lncRNA-H19 expression. LncRNA-H19 overexpression resulted in a significant decline in the level of miR-199a-5p under both normoxia and H/SD conditions. However, its inhibition elevated the expression of miR-199a-5p. These results reflected that lncRNA-H19 functioned as a ceRNA and modulated the expression of miR-199a-5p to improve MSCs survival and angiogenic capacity.

## Conclusion

In summary, we demonstrated the role of lncRNA-H19 in enhancing MSCs survival and angiogenic capacity in vitro, and identified a potential ceRNA network by which lncRNA-H19 acted as a molecular sponge for miR-199a-5p to regulate the expression of VEGFA in MSCs. We showed that miR-199a-5p was a target of lncRNA-H19. Further exploration of the pleiotropic effects of lncRNA-H19 and the crosstalk between lncRNA-H19 and miR-199a-5p will provide new insights for developing new strategies to improve the therapeutic efficacy based on MSCs transplantation.
